# Typhoid Fever in South Africa in an Endemic HIV Setting

**DOI:** 10.1371/journal.pone.0164939

**Published:** 2016-10-25

**Authors:** Karen H. Keddy, Arvinda Sooka, Anthony M. Smith, Alfred Musekiwa, Nomsa P. Tau, Keith P. Klugman, Frederick J. Angulo, GERMS-SA

**Affiliations:** 1 Centre for Enteric Diseases, National Institute for Communicable Diseases, Johannesburg, South Africa; 2 Faculty of Health Sciences, University of the Witwatersrand, Johannesburg, South Africa; 3 International Emerging Infections Programme, South Africa Global Disease Detection Centre, Centers for Disease Control and Prevention, Pretoria, South Africa; 4 Bill and Melinda Gates Foundation, Seattle, WA, United States of America; 5 Division of Global Health Protection, Center for Global Health, Centers for Disease Control and Prevention, Atlanta, GA, United States of America; Indian Institute of Science, INDIA

## Abstract

**Background:**

Typhoid fever remains an important disease in Africa, associated with outbreaks and the emerging multidrug resistant *Salmonella enterica* serotype Typhi (*Salmonella* Typhi) haplotype, H58. This study describes the incidence of, and factors associated with mortality due to, typhoid fever in South Africa, where HIV prevalence is high.

**Methods and Findings:**

Nationwide active laboratory-based surveillance for culture-confirmed typhoid fever was undertaken from 2003–2013. At selected institutions, additional clinical data from patients were collected including age, sex, HIV status, disease severity and outcome. HIV prevalence among typhoid fever patients was compared to national HIV seroprevalence estimates. The national reference laboratory tested *Salmonella* Typhi isolates for antimicrobial susceptibility and haplotype. Unadjusted and adjusted logistic regression analyses were conducted determining factors associated with typhoid fever mortality. We identified 855 typhoid fever cases: annual incidence ranged from 0.11 to 0.39 per 100,000 population. Additional clinical data were available for 369 (46.8%) cases presenting to the selected sites. Among typhoid fever patients with known HIV status, 19.3% (29/150) were HIV-infected. In adult females, HIV prevalence in typhoid fever patients was 43.2% (19/44) versus 15.7% national HIV seroprevalence (P < .001); in adult males, 16.3% (7/43) versus 12.3% national HIV seroprevalence (P = .2). H58 represented 11.9% (22/185) of *Salmonella* Typhi isolates tested. Increased mortality was associated with HIV infection (AOR 10.7; 95% CI 2.3–50.3) and disease severity (AOR 9.8; 95% CI 1.6–60.0) on multivariate analysis.

**Conclusions:**

Typhoid fever incidence in South Africa was largely unchanged from 2003–2013. Typhoid fever mortality was associated disease severity. HIV infection may be a contributing factor. Interventions mandate improved health care access, including to HIV management programmes as well as patient education. Further studies are necessary to clarify relationships between HIV infection and typhoid fever in adults.

## Introduction

Typhoid fever, infection with *Salmonella enterica* serotype Typhi (*Salmonella* Typhi), remains an important infectious disease in developing countries; the estimated annual disease burden is 10 to 13 million cases [1] with 190 000 deaths globally [2]. Typhoid fever mortality in Africa has been estimated at 7.2 per 100 000 person-years, double that of south Asia and 25–70 times that of the rest of the world in a recent systematic review [1]. Typhoid fever appears particularly problematic in emerging economies, with high burdens reported from Kenya [3], Pakistan, India and Indonesia [3], where disease is associated with urban informal settlements [3,4]. Numerous data gaps remain regarding typhoid fever in low and middle income countries including factors associated with death among typhoid fever patients [5]. The association of HIV infection with typhoid fever also remains unclear: an increased risk of acquiring typhoid fever among HIV-infected persons was described in Peru [6], whereas a Tanzanian study found HIV infection was associated with a decreased risk of typhoid fever [7]. HIV infection is a particular public health problem in South Africa where 15% of the population ≥15 years of age were HIV-infected in 2013 [8].

Recently, a multidrug resistant haplotype of *Salmonella* Typhi, H58, has emerged in South East Asia [9–11], and Africa [12,13]. This emergence may be related to increased virulence or selective pressures created by antimicrobial use [14]. We report on the incidence of typhoid fever in South Africa from 2003 to 2013, the clinical and microbiological features of typhoid fever that may drive mortality, including association with HIV infection and the prevalence of *Salmonella* Typhi H58.

## Methods

### Surveillance

Nationwide active laboratory-based surveillance for culture-confirmed typhoid fever, (isolation of *Salmonella* Typhi from a normally sterile site), was performed by the Centre for Enteric Diseases (CED) of the National Institute for Communicable Diseases in South Africa from 2003 through 2013, as previously described [15]. Specifically, we requested that all diagnostic laboratories in South Africa send all *Salmonella* Typhi isolates to CED and performed audits to identify additional laboratory-confirmed cases of typhoid fever for which *Salmonella* Typhi isolates were not received using the Central Data Warehouse (CDW), a national databank derived from the laboratory information system storing basic demographic and laboratory data for all typhoid fever cases diagnosed by the National Health Laboratory Service [15] (active case finding). Ethical approval for this study was granted by the Human Research Ethics Committee of the University of the Witwatersrand (M110601).

At 25 hospitals selected to represent major centres in the nine South African provinces, additional information was collected from medical records of typhoid fever patients through GERMS-SA, a national surveillance network sharing resources to characterise diseases of public health importance in South Africa. Written informed consent from the patient or (care provider, in the case of minors) was obtained for those patients for whom interviews were conducted. Where clinical data was obtained solely from clinical record review, no written informed consent was obtained from the patient. Patient records could not be anonymised as patient information was required to cross reference each case with those cases that were notified through audit of the NHLS CDW, to avoid duplicating case counts. Patient records were reviewed for clinical presentation, HIV infection and other co-morbidities, and outcome (survival or death). A patient was considered to have co-morbid disease if they had any of the following diagnoses: tuberculosis, malignancy, autoimmune disease, renal disease, liver failure, diabetes mellitus, protein energy malnutrition (children) or congested cardiac failure. A Pitt bacteraemia score (PBS) was determined for patients with typhoid fever by assigning values between 0 and 2 for presence of fever or hypopyrexia, mechanical ventilation and hypotension or 0 and 4 for cardiac arrest and mental status. Higher scores corresponded with increased severity of illness [15,16].

HIV prevalence among male and female typhoid fever patients ≥15 years of age were compared to national HIV seroprevalence estimates from the 2008 Actuarial Society of South Africa (ASSA) [8].

### Laboratory

All clinical diagnostic laboratories in South Africa were requested to submit *Salmonella* Typhi isolates to CED for confirmation and further characterisation in accordance with GERMS-SA network guidelines. At CED, *Salmonella* serotype was confirmed according to the White-Kaufmann-Le Minor scheme and antimicrobial susceptibility to ampicillin, trimethoprim-sulfamethoxazole, chloramphenicol, tetracycline, ciprofloxacin and ceftriaxone was determined using E-tests, according to the manufacturer’s instructions (BioMérieux, Marcy-l’Étoile, France). Multidrug resistance (MDR) was defined as resistance to three or more of these antimicrobials.

The presence of the H58 haplotype was confirmed using conventional PCR [17].

### Statistical analysis

Typhoid fever incidence calculations used national population estimates from ASSA [8]. Univariate and multivariate logistic regression were used to determine factors (age, sex, HIV status, PBS, other comorbidities, multidrug resistance and H58 haplotype) associated with mortality among typhoid fever patients. Analyses were performed using Stata version 13 (StataCorp Limited, College Station, TX, USA). Two-sided P values of < .05 were considered significant throughout. To assess changes in typhoid fever mortality over time, corresponding to changes in the proportion of the population of HIV-infected persons with access to antiretroviral treatment [8], we defined early (2003–2005), middle (2006–2010) and late (2011–2013) study periods. The Fisher Exact test and Chi-squared (in adult women) test were used to compare HIV prevalence among typhoid fever patients and national seroprevalence estimates.

## Results

### Surveillance

**Fig 1:**
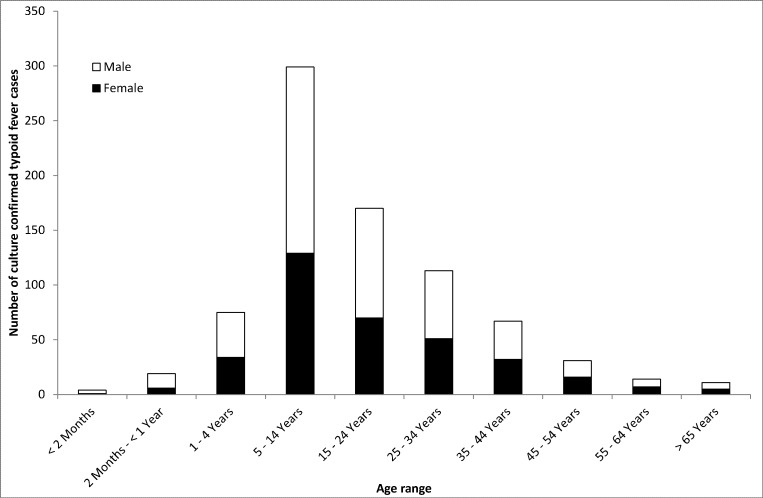
Number of culture-confirmed typhoid fever cases (N = 803) by age range and sex in South Africa, 2003–2013.

From 2003 to 2013, we ascertained 855 cases of culture-confirmed typhoid fever in South Africa: 67 (7.8%) were identified on audit of the CDW: the number of cases annually ranging from 48 to 187. With the exception of increases in culture-confirmed typhoid cases in 2005 and 2006, the incidence in South Africa was largely unchanged from 2003 to 2013, ranging from 0.11 per 100,000 population in 2013 to 0.39 per 100,000 population in 2005 (Table 1). Age was available for 814 cases, ranging from 0 days (birth) to 81 years with a median of 15 years. Sex was available for 841 cases; 470 (55.9%) were male. Sex and age were available for 803 cases (Fig 1). Among 855 typhoid fever cases, *Salmonella* Typhi was isolated from blood in 816 (95.4%), CSF in 4 (0.5%) and other body sites in 35 (4.1%) cases.

**Table 1:** Number of cases and incidence rates per 100,000 population of culture-confirmed typhoid fever cases in South Africa 2003–2013 (N = 855).

Year	Number of culture-confirmed typhoid fever cases	South African Population*	Incidence rate of culture-confirmed typhoid fever cases
2003	60	47150661	0.13
2004	72	47676795	0.15
2005	187	48155945	0.39
2006	114	48605035	0.23
2007	66	49034069	0.13
2008	67	49459134	0.14
2009	58	49905065	0.12
2010	62	50371513	0.12
2011	64	50840589	0.13
2012	48	51304466	0.09
2013	57	51759127	0.11

*Data derived from ASSA tables [8].

### Laboratory

Isolates were received at CED from 788 (92.2%) of the 855 cases: 760 (96.4%) were viable and confirmed as *Salmonella* Typhi. Antimicrobial susceptibility testing on 758 (99.7%) indicated 175 (23.1%) were resistant to ampicillin, 165 (21.8%) to co-trimoxazole, 91 (12.0%) to chloramphenicol, 111 (14.6%) to tetracycline, 69 (9.1%) resistant to ciprofloxacin and 1 (0.1%) to ceftriaxone (two isolates died before susceptibilities could be confirmed). Multidrug resistance was recorded in 158 (20.8%). *Salmonella* Typhi H58 represented 22 (11.9%) of 185 isolates tested; 14/22 (63.6%) H58 isolates were multidrug resistant compared with 26/163 (16.0%) non-H58 isolates (P < .001).

### Selected sites

Additional clinical data including outcome (recovery or death) were available for 369 (46.8%) cases presenting to the selected sites. Presence or absence of fever was recorded for 215 (59.1%) cases; 130 (60.5%) presented with fever (temperature ≥38°C) and 18 (8.4%) were hypopyrexial (temperature <36.5°C). Of the 196 cases with sufficient clinical information to derive a PBS, 12 (6.1%) had a PBS ≥4; patients with PBS ≥4 reported later to hospital compared with patients with PBS <4 (data not shown). HIV status at time of typhoid fever diagnosis was recorded for 150 (41.2%) cases; 29 (19.3%) were HIV-infected (Table 2). Record review suggested no differences in patient management between 29 HIV-infected patients compared with 121 HIV uninfected patients. Among 63 persons <15 years of age with recorded HIV status, 3 (4.8%) were HIV-infected. Among 87 persons ≥ 15 years of age with recorded HIV status, 26 (29.9%) were HIV-infected. The proportion of typhoid fever patients ≥15 years of age who were HIV-infected at the time of typhoid fever diagnosis was 36.4% (8/22) in 2003–2005, 28.1% (9/32) in 2006–2010, and 27.3% (9/33) in 2011–2013. Among female patients ≥15 years of age with recorded HIV status, 19/44 (43.2%) were HIV-infected at time of typhoid fever diagnosis versus 7/43 (16.3%) males (p = .009).

**Table 2:** Clinical and laboratory characteristics associated with patients with culture-confirmed typhoid fever in South Africa (N = 369), 2003–2013, based on data from sentinel sites.

Characteristic	Total n (%)
Age	361*
< 2 Months	1 (0.3)
2 Months—< 1 Year	10 (2.8)
1–4 Years	38 (10.5)
5–14 Years	132 (36.6)
15–24 Years	84 (23.3)
25–34 Years	46 (12.7)
35–44 Years	28 (7.8)
45–54 Years	11 (3.0)
55–64 Years	6 (1.6)
> 65 Years	5 (1.4)
Sex	367*
Male	196 (53.4)
Female	171 (46.6)
Outcome	237*
Death	16 (6.8)
Recovery	221 (93.2)
HIV status	150*
Uninfected	121 (80.7)
Infected	29 (19.3)
Antiretroviral access period	369*
Early 2003–2005	141 (38.3)
Mid 2006–2009	140 (37.9)
Late 2010–2013	88 (23.8)
Pitt bacteraemia score (severity of illness)	196*
<4	184 (93.9)
≥4	12 (6.1)
Other comorbidity	362*
No	336 (92.8)
Yes	26 (7.2)
Multidrug resistance	330*
No	236 (71.5)
Yes	94 (28.5)
*Salmonella* Typhi haplotype	176*
Non-H58	155 (88.1)
H58	21 (11.9)

*Total excludes those patients for whom results were unknown.

According to ASSA estimates from 2003 to 2013, average national HIV seroprevalence in females ≥15 years of age was 15.7%; female typhoid fever patients ≥15 years of age had a significantly higher HIV seroprevalence (19/44; 43.2%) than national estimates for females ≥15 years of age (15.7%) (p < .001). Average national HIV seroprevalence from 2003 to 2013 in males ≥15 years of age was 12.3%; male typhoid fever patients ≥15 years of age had a higher HIV seroprevalence (7/43; 16.3%) than national estimates for males ≥15 years of age (12.3%), although this was not significant (p = .2).

The presence or absence of co-morbidities for typhoid cases was determined from medical records available 362 (98.1%) of 369 cases available from these sites; 26 (7.2%) had co-morbidities including diabetes mellitus, malignancy, protein-energy malnutrition, congestive cardiac failure and hepatic failure. No patients, including HIV-infected patients, had other bacterial co-infections. Eight HIV-infected patients refused hospital treatment; further clinical information was unavailable from these patients.

Final patient outcome (hospital discharge or death) was available for 237/369 (65.1%) cases from these sites; 16 (6.8%) died (Table 2). Among the patients who died, sex was known for 15; 7 (46.7%) were male. The ages of these 16 patients ranged from 7 to 65 years, with a median of 19.5 years. Of the 237 cases with final hospital outcome data, *Salmonella* Typhi was isolated from blood or CSF in 232 cases; 196 of these had a recorded PBS (median PBS 1; range 0–7), of whom 12 (6.1%) had a PBS ≥4.

Final hospital outcome data were known for 55 of 158 (34.8%) typhoid fever patients infected with multidrug resistant *Salmonella* Typhi; 5 (9.1%) of whom died. Outcome was known for 19 (90.5%) of the 21 typhoid fever patients infected with H58, of whom one (5.3%) died.

On unadjusted analysis, age ≥15 years (Odds Ratio [OR] 4.1; 95% Confidence Interval [CI] 1.1–14.9), HIV infection (OR 11.3; 95% CI 3.0–42.4), and PBS ≥4 (OR 10.8; CI 2.9–39.5) were associated with death among typhoid fever patients (Table 3). On adjusted analysis, HIV infection (Adjusted OR [AOR] 10.8; 95% CI 2.3–50.3) and PBS ≥4 (AOR 9.8; 95% CI 1.6–60.0) remained associated with mortality. Neither multidrug resistance nor *Salmonella* Typhi H58 was associated with mortality among typhoid fever patients (Table 3); there were additionally no differences between HIV-uninfected and HIV-infected individuals in the occurrence of multidrug resistance *Salmonella* Typhi or the isolation of *Salmonella* Typhi H58 (P = 0.5and P = 0.3, respectively).

**Table 3:** Univariate and multivariate analysis of risk factors associated with mortality in patients with *Salmonella* Typhi in South Africa, 2003–2013.

	Unadjusted analysis			Adjusted analysis		
Characteristic	OR	(95% CI)	*P*	AOR	(95% CI)	*P*
Age						
<15 years	1	-	-	1	-	-
≥15 years	4.1	(1.1–14.9)	.03	2.0	(0.2–19.3)	.6
Sex						
Male	1	-	-			
Female	1.4	(0.5–3.8)	.5			
HIV status						
Uninfected	1	-	-	1	-	-
Infected	11.3	(3.0–42.4)	< .001	10.8	(2.3–50.3)	.002
Antiretroviral access period						
Early 2003–2005	1	-	-			
Mid 2006–2009	1.7	(0.6–5.4)	.3			
Late 2010–2013	0.4	(0.1–2.3)	.3			
Pitt bacteraemia score						
<4	1	-	-	1	-	-
≥4	10.8	(2.9–39.5)	< .001	9.8	(1.6–60.0)	.01
Other co-morbid conditions						
No	1	-	-			
Yes	2.1	(0.5–8.0)	.2			
Multidrug resistance						
No	1	-	-			
Yes	1.7	(0.6–5.3)	.3			
*Salmonella* Typhi haplotype						
Non-H58	1	-	-			
H58	0.7	(0.09–5.8)	.8			

OR, odds ratio; CI, confidence interval; AOR, adjusted odds ratio

## Discussion

Typhoid fever remains an important public health challenge in South Africa [18–20]. Incidence rates of culture-confirmed typhoid fever were between 0.11 and 0.39 per 100,000 population; peaking in 2005 and 2006, due to typhoid outbreaks of over 600 clinically diagnosed cases, of which approximately 160 were culture-confirmed and captured in our surveillance [19].

Factors associated with mortality among persons with typhoid fever on multivariate analysis were HIV-infection and disease severity as measured by PBS (Table 3). When we reviewed individual patient records from the sentinel sites, no differences were identified between the management of HIV-infected and uninfected patients: i.e. all patients were appropriately and timeously treated, based on a positive culture result of invasive *Salmonella* Typhi at the presenting hospital. Independently of HIV status, PBS score was significantly associated with mortality. Specifically, in our series, late access to health care appeared critical in contributing to mortality due to severity of illness in typhoid fever patients, rather than belated diagnosis and treatment delays in hospital or inappropriate treatment.

Among the clinical records we reviewed, only HIV-infected typhoid fever patients refused hospital treatment. This, and the observation that typhoid fever patients may delay accessing health care, suggest patient and population education is paramount in managing typhoid fever from a public health and an individual patient perspective.

Our finding that 16.3% of adult male patients versus 43.2% of adult females with typhoid fever were HIV-infected may be related to the antenatal testing programme for HIV status in pregnant women within the country [21]: most South African women who have borne children would know their HIV status, which would be recorded on hospital admission, in contrast to South African men. Sex, moreover, was not significantly associated with mortality. Previously we observed a female predominance among HIV-infected adults with systemic shigellosis, which included patients from selected and non-selected sites and was ascribed to the burden of childcare of children with *Shigella* diarrhoea [15]. Here, no general female predominance was observed: 53.4% culture-confirmed cases in sentinel sites were male: the marked HIV predominance recorded among women in this series may potentially be indicative of testing biases. HIV infection is also more common in women of child-bearing age than in men [8,21], thus, a combination of factors, both clinical and due to sampling methods, may have influenced the numbers of HIV-infected women presenting with typhoid fever, as limited numbers of patients from the sentinel sites had HIV status determined.

HIV prevalence observed among women and men ≥15 years of age with typhoid fever, was higher than the comparable national seroprevalence, significantly so in women [8], suggesting HIV-infected adults may be at greater risk of acquiring typhoid fever. This apparent predominance contrasts observations by Crump *et al* [7], who found HIV-infected adolescents and adults less likely to acquire typhoid fever compared with HIV-uninfected patients. This earlier study was a clinically-based fever study: only patients with documented fever >38°C were included. Our study used laboratory-based surveillance enrolling all patients with culture-confirmed disease, irrespective of clinical characteristics, including fever. Approximately 40% of our patients were hypopyrexial or apyrexial on admission, possibly contributing to these observed differences. As the immune response is necessary for the development of fever [22], if this is defective, it is feasible the classic fever curves associated with typhoid fever may not be observed. Moreover, functional CD4 cells are necessary to combat typhoid fever in healthy adults [23] and transient defects in these cells have previously been associated with opportunistic typhoid fever infection [24].

Undefined parameters may nonetheless make women more susceptible to typhoid fever: Khan *et al* described a series from Durban, South Africa, in which women were more likely to have severe disease, although they excluded HIV status in the analysis [25]. Other literature series examining the association of HIV and typhoid fever have looked at populations where HIV is predominantly a male disease, in men who have sex with men [6,26], where sexual practices may constitute an additional risk factor, or the male-to-female ratio for typhoid fever was not reported [7]. Not all reports from African countries have highlighted a female predominance [4,27], although increased numbers of women were reported from large outbreaks of typhoid fever in Zimbabwe [28] and Malawi [29]: HIV status was not documented in these reports. In Zambia, male-to-female ratios were equal in a large outbreak from 2010 to 2012, but most cases were reported in children less than 15 years of age [12].

In children, typhoid fever is typically associated with the 5 to 15 year age group in South Africa [18], in which the HIV prevalence was calculated at 3% in 2003 [8]; in the absence of ART, few HIV-infected children survive beyond 5 years of age. It is likely in this age group, very few HIV-infected children with typhoid fever would have unknown HIV status, given current testing recommendations in the country [30]. Most children with typhoid fever in this series under five years of age would have benefited from the perinatal antiretroviral programme introduced in 2004 [31], very few HIV-infected children would be expected among this age group, in which typhoid fever incidence is also low. It is therefore unsurprising that typhoid fever was not significantly associated with HIV infection in children under 15 years of age.

The presence of multidrug resistant (MDR) *Salmonella* Typhi H58 in South Africa has previously been reported [32]. This virulent, MDR clade affected both HIV-infected and uninfected patients equally, irrespective of these two groups of patients’ susceptibility to typhoid fever. This virulent strain appears not to be specifically adapted to a vulnerable population group, as has been reported with *Salmonella* Typhimurium ST313 in Africa [33], including an association with *Salmonella* meningitis in South Africa [34], but probably emerged as a result of global population movements and wide-spread use and misuse of antimicrobial therapy [32].

This study had limitations. Primarily, missing data for certain clinical parameters may have affected the data analyses and results. Secondly, not all cases of typhoid fever over the study period were included: the majority of cases related to the 2005 typhoid fever outbreak did not have blood cultures done due to resource constraints [19]. In non-outbreak years, cases may similarly have been missed, thus burden of disease estimates could not be calculated. A lack of robustness of the data with year-on-year variation in case numbers and incompleteness of the HIV results meant that imputation of data for patients with unknown HIV status could lead to biased inference. From 2004, ART was rolled out in South Africa [31]. We attempted to overcome the data insufficiencies regarding HIV status through analysing the data based on early, mid and late ART periods, showing no association between HIV and typhoid fever in children, possibly due to decreasing HIV seroprevalence following the introduction of ART [30] and the preponderance of children aged 5 to 14 years with typhoid fever [18]. Limited numbers of cases with HIV results and the increased prevalence of HIV infection in adults and in women in particular may account for apparent associations between HIV-infected women and typhoid fever. HIV seroprevalence in adults was nonetheless greater than the overall prevalence of HIV-infected adults in South Africa [8]: the role of HIV as a risk factor for the development of typhoid fever still needs further exploration.

## Conclusions

In summary, typhoid fever in South Africa remains a public health challenge. Persons with typhoid fever who are HIV-infected or severely ill are more prone to mortality. Disease severity may be affected by access to health care and treatment delays; multidrug resistance and H58 haplotype were not associated with mortality. *Salmonella* Typhi H58 has not specifically emerged in Africa in response to the HIV pandemic, but has probably expanded as a clone in association with population movements and antimicrobial usage practices on the African continent. Public health interventions should include patient and population education and enhanced management of HIV infection, including testing patients with typhoid fever, who may be at risk for HIV infection, in order that they may benefit from national treatment plans.
